# Dimethyl 4-[3-(4-meth­oxy­phen­yl)-1-phenyl-1*H*-pyrazol-4-yl]-2,6-dimethyl-1,4-dihydro­pyridine-3,5-dicarboxyl­ate dihydrate

**DOI:** 10.1107/S1600536812027936

**Published:** 2012-06-27

**Authors:** Hoong-Kun Fun, Chin Wei Ooi, B. Garudachari, Kammasandra Nanjunda Shivananda, Arun M. Isloor

**Affiliations:** a X-ray Crystallography Unit, School of Physics, Universiti Sains Malaysia, 11800 USM, Penang, Malaysia; b Medicinal Chemistry Laboratory, Department of Chemistry, National Institute of Technology Karnataka, Surathkal, Mangalore 575 025, India; c Schulich Faculty of Chemistry, Technion Israel Institute of Technology, Haifa 32000, Israel

## Abstract

In the title compound, C_27_H_27_N_3_O_5_·2H_2_O, the dihydro­pyridine ring adopts a flattened boat conformation. The central pyrazole ring is essentially planar [maximum deviation of 0.003 (1) Å] and makes dihedral angles of 50.42 (6) and 26.44 (6)° with the benzene rings. In the crystal, mol­ecules are linked *via* N—H⋯O, O—H⋯O, O—H⋯N and C—H⋯O hydrogen bonds into two-dimensional networks parallel to the *bc* plane. The crystal structure is further consolidated by weak C—H⋯π inter­actions.

## Related literature

For details and applications of pyrazoles, see: Buhler & Kiowski (1987 ▶); Isloor *et al.* (2000 ▶, 2009 ▶); Isloor (2011 ▶); Vijesh *et al.* (2011 ▶); Vo *et al.* (1995 ▶). For the preparation of the compound, see: Trivedi *et al.* (2011 ▶). For ring conformations, see: Cremer & Pople (1975 ▶). For related structures, see: Fun *et al.* (2011 ▶, 2012 ▶). For bond-length data, see: Allen *et al.* (1987 ▶). For the stability of the temperature controller used in the data collection, see: Cosier & Glazer (1986 ▶).
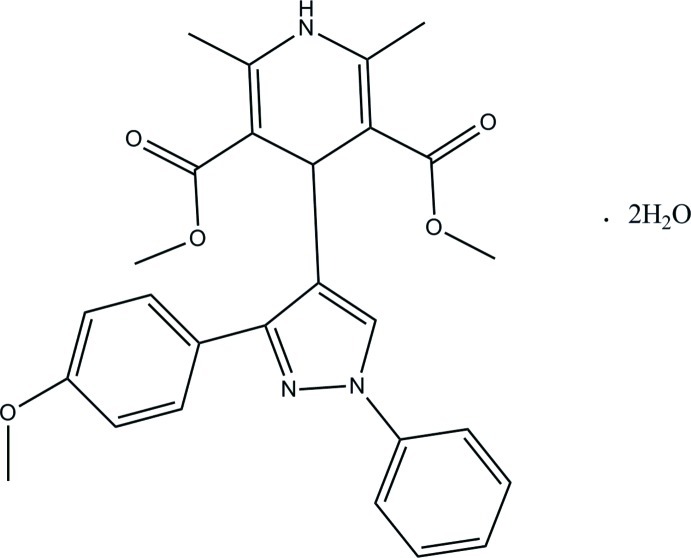


## Experimental

### 

#### Crystal data

C_27_H_27_N_3_O_5_·2H_2_O
*M* *_r_* = 509.55
Monoclinic, 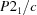

*a* = 14.1279 (9) Å
*b* = 11.6313 (7) Å
*c* = 15.3780 (9) Å
β = 93.358 (1)°
*V* = 2522.7 (3) Å^3^
*Z* = 4
Mo *K*α radiation
μ = 0.10 mm^−1^
*T* = 100 K
0.34 × 0.17 × 0.14 mm

#### Data collection

Bruker APEX DUO CCD area-detector diffractometer
Absorption correction: multi-scan (*SADABS*; Bruker, 2009 ▶) *T*_min_ = 0.968, *T*_max_ = 0.987
28375 measured reflections
7347 independent reflections
5683 reflections with *I* > 2σ(*I*)
*R*_int_ = 0.036

#### Refinement

*R*[*F* ^2^ > 2σ(*F* ^2^)] = 0.042
*wR*(*F* ^2^) = 0.131
*S* = 1.04
7347 reflections
359 parameters
H atoms treated by a mixture of independent and constrained refinement
Δρ_max_ = 0.43 e Å^−3^
Δρ_min_ = −0.24 e Å^−3^

### 

Data collection: *APEX2* (Bruker, 2009 ▶); cell refinement: *SAINT* (Bruker, 2009 ▶); data reduction: *SAINT*; program(s) used to solve structure: *SHELXTL* (Sheldrick, 2008 ▶); program(s) used to refine structure: *SHELXTL*; molecular graphics: *SHELXTL*; software used to prepare material for publication: *SHELXTL* and *PLATON* (Spek, 2009 ▶).

## Supplementary Material

Crystal structure: contains datablock(s) global, I. DOI: 10.1107/S1600536812027936/sj5244sup1.cif

Structure factors: contains datablock(s) I. DOI: 10.1107/S1600536812027936/sj5244Isup2.hkl

Supplementary material file. DOI: 10.1107/S1600536812027936/sj5244Isup3.cml

Additional supplementary materials:  crystallographic information; 3D view; checkCIF report

## Figures and Tables

**Table 1:** Hydrogen-bond geometry (Å, °) *Cg*1 and *Cg*2 are the centroids of the pyrazole (N2/N3/C6–C8) and benzene (C15–C20) rings, respectively.

*D*—H⋯*A*	*D*—H	H⋯*A*	*D*⋯*A*	*D*—H⋯*A*
N1—H1*N*1⋯O2*W*	0.904 (18)	2.001 (18)	2.9020 (14)	175.2 (15)
O1*W*—H2*W*1⋯N3	0.90 (2)	2.05 (2)	2.9449 (14)	174 (2)
O2*W*—H2*W*2⋯O1*W* ^i^	0.98 (3)	1.84 (3)	2.7986 (15)	166 (2)
O2*W*—H1*W*2⋯O3^ii^	0.90 (3)	1.91 (3)	2.8074 (14)	174 (2)
O1*W*—H1*W*1⋯O2*W* ^iii^	0.87 (3)	2.06 (3)	2.9274 (15)	178 (2)
C20—H20*A*⋯O1*W* ^iv^	0.95	2.44	3.2980 (16)	151
C13—H13*A*⋯*Cg*2^v^	0.95	2.90	3.6957 (14)	142
C18—H18*A*⋯*Cg*1^vi^	0.95	2.63	3.3682 (15)	135
C25—H25*A*⋯*Cg*2^i^	0.98	2.87	3.7231 (14)	146
C27—H27*C*⋯*Cg*1^v^	0.98	2.62	3.5720 (17)	164

Symmetry codes: (i) 
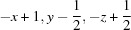
; (ii) 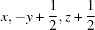; (iii) 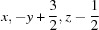; (iv) 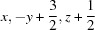; (v) 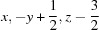; (vi) 
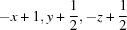
.

**Fig. 1.**
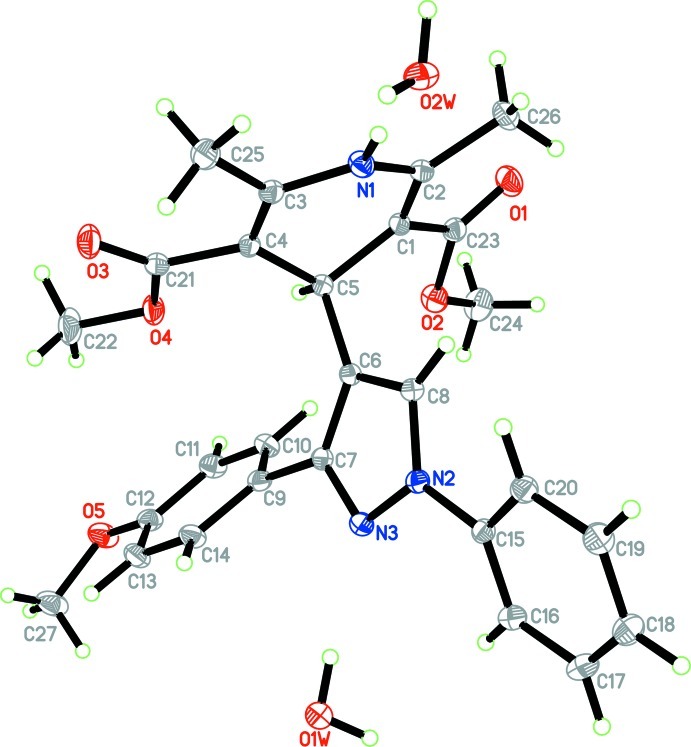
The molecular structure of the title compound, showing 50% probability displacement ellipsoids and the atom-numbering scheme.

**Fig. 2.**
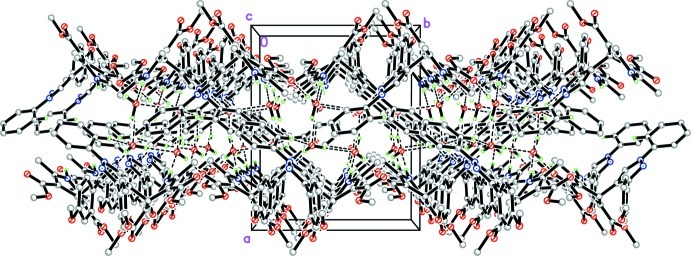
The crystal packing of the title compound, viewed along the c axis. H atoms not involved in the intermolecular interactions (dashed lines) have been omitted for clarity.

## supplementary crystallographic information

### Comment

In the recent years, pyrazoles and their derivatives have attracted medicinal chemists because of their varied biological properties such as anti-microbial (Isloor *et al.*, 2009), analgesic (Isloor *et al.*, 2000; Isloor, 2011) and anti-inflammatory (Vijesh *et al.*, 2011) activities. They are used most frequently as cardiovascular agents for the treatment of hypertension (Buhler & Kiowski, 1987). A number of dihydropyridine (DHP) derivatives are employed as potential drug candidates for the treatment of congestive heart failure (Vo *et al.*, 1995). In view of this potential biological importance, we have synthesised the title DHP compound and report its structure here.

The asymmetric unit of the title compound (Fig. 1), contains one dimethyl 4-[3-(4-methoxyphenyl)-1-phenyl-1H-pyrazol-4-yl]-2,6-dimethyl-1,4-dihydropyridine-3,5-dicarboxylate molecule and two water molecules. The dihydropyridine (N1/C1–C5) ring adopts a flattened boat conformation with puckering parameters (Cremer & Pople, 1975) Q = 0.3369 (12) Å, θ = 107.1 (2)°, and φ = 1.5 (2)°. The central pyrazole ring (N2/N3/C6–C8) is essentially planar [maximum deviation of 0.003 (1) Å at atoms N2 and C8] and makes dihedral angles of 50.42 (6)° and 26.44 (6)°, respectively, with the benzene rings (C9–C14 & C15–C20). The bond lengths (Allen *et al.*, 1987) and angles are within normal ranges and are comparable to those found in related structures (Fun *et al.*, 2011, 2012).

In the crystal structure (Fig. 2), the molecules are linked *via* intermolecular N1—H1N1···O2W, O1W—H1W1···O2W, O1W—H2W1···N3, O2W—H1W2···O3, O2W—H2W2···O1W and C20—H20A···O1W hydrogen bonds (Table 1) into two-dimensional networks parallel to the *bc* plane. The crystal structure is further consolidated by weak C—H···π interactions (Table 1), involving the centroids of the pyrazole ring (N2/N3/C6–C8; *Cg*1) and benzene ring (C15–C20; *Cg*2).

### Experimental

3-(4-Methoxyphenyl)-1-phenyl-1*H*-pyrazole-4-carbaldehyde (0.20 g, 0.0007 mol), acetyl acetone (0.17 g, 0.0015 mol) and ammonium acetate (0.064 g, 0.00084 mol) in ethanol (10 ml) were refluxed for 5 h. After the completion of the reaction, the reaction mixture was concentrated and poured into crushed ice. The precipitated product was filtered and washed with water. The resulting solid was recrystallized from ethanol. Yield: 0.28 g, 82.3%; *M.p.* 393–395 K. (Trivedi *et al.*, 2011).

### Refinement

All N and O bound H atoms were located from the difference map and were refined freely [N–H = 0.904 (18) Å and O–H = 0.87 (3)–0.98 (2) Å]. The remaining H atoms were positioned geometrically and refined using a riding model with *U*_iso_(H) = 1.2 or 1.5*U*_eq_(C) (C–H = 0.9500, 0.9800 and 1.0000 Å). A rotating group model was applied to the methyl groups.

### Crystal data

**Table:**

C_27_H_27_N_3_O_5_·2H_2_O	*F*(000) = 1080
*M**_r_* = 509.55	*D*_x_ = 1.342 Mg m^−^^3^
Monoclinic, *P*2_1_/*c*	Mo *K*α radiation, λ = 0.71073 Å
Hall symbol: -P 2ybc	Cell parameters from 7658 reflections
*a* = 14.1279 (9) Å	θ = 2.3–30.0°
*b* = 11.6313 (7) Å	µ = 0.10 mm^−^^1^
*c* = 15.3780 (9) Å	*T* = 100 K
β = 93.358 (1)°	Block, yellow
*V* = 2522.7 (3) Å^3^	0.34 × 0.17 × 0.14 mm
*Z* = 4	

### Data collection

**Table:**

Bruker APEX DUO CCD area-detector diffractometer	7347 independent reflections
Radiation source: fine-focus sealed tube	5683 reflections with *I* > 2σ(*I*)
Graphite monochromator	*R*_int_ = 0.036
φ and ω scans	θ_max_ = 30.1°, θ_min_ = 1.4°
Absorption correction: multi-scan (*SADABS*; Bruker, 2009)	*h* = −19→19
*T*_min_ = 0.968, *T*_max_ = 0.987	*k* = −16→13
28375 measured reflections	*l* = −21→21

### Refinement

**Table:**

Refinement on *F*^2^	Primary atom site location: structure-invariant direct methods
Least-squares matrix: full	Secondary atom site location: difference Fourier map
*R*[*F*^2^ > 2σ(*F*^2^)] = 0.042	Hydrogen site location: inferred from neighbouring sites
*wR*(*F*^2^) = 0.131	H atoms treated by a mixture of independent and constrained refinement
*S* = 1.04	*w* = 1/[σ^2^(*F*_o_^2^) + (0.0713*P*)^2^ + 0.6924*P*] where *P* = (*F*_o_^2^ + 2*F*_c_^2^)/3
7347 reflections	(Δ/σ)_max_ < 0.001
359 parameters	Δρ_max_ = 0.43 e Å^−^^3^
0 restraints	Δρ_min_ = −0.24 e Å^−^^3^

### Special details

**Table:**

Experimental. The crystal was placed in the cold stream of an Oxford Cryosystems Cobra open-flow nitrogen cryostat (Cosier & Glazer, 1986) operating at 100 (1) K.
Geometry. All e.s.d.'s (except the e.s.d. in the dihedral angle between two l.s. planes) are estimated using the full covariance matrix. The cell e.s.d.'s are taken into account individually in the estimation of e.s.d.'s in distances, angles and torsion angles; correlations between e.s.d.'s in cell parameters are only used when they are defined by crystal symmetry. An approximate (isotropic) treatment of cell e.s.d.'s is used for estimating e.s.d.'s involving l.s. planes.
Refinement. Refinement of *F*^2^ against ALL reflections. The weighted *R*-factor *wR* and goodness of fit *S* are based on *F*^2^, conventional *R*-factors *R* are based on *F*, with *F* set to zero for negative *F*^2^. The threshold expression of *F*^2^ > σ(*F*^2^) is used only for calculating *R*-factors(gt) *etc*. and is not relevant to the choice of reflections for refinement. *R*-factors based on *F*^2^ are statistically about twice as large as those based on *F*, and *R*- factors based on ALL data will be even larger.

### Fractional atomic coordinates and isotropic or equivalent isotropic displacement parameters (Å^2^)

**Table:**

	*x*	*y*	*z*	*U*_iso_*/*U*_eq_	
O1	−0.00103 (7)	0.62548 (9)	0.37770 (6)	0.0226 (2)	
O1W	0.41969 (8)	0.86761 (10)	−0.00494 (6)	0.0234 (2)	
O2	0.03130 (6)	0.66323 (8)	0.23892 (6)	0.01912 (19)	
O2W	0.38758 (7)	0.38977 (8)	0.53450 (6)	0.0195 (2)	
O3	0.28443 (7)	0.24426 (8)	0.14770 (6)	0.0219 (2)	
O4	0.18314 (7)	0.37519 (8)	0.08778 (6)	0.0200 (2)	
O5	0.08804 (7)	0.56032 (9)	−0.20791 (6)	0.0225 (2)	
N1	0.25743 (8)	0.42928 (9)	0.38442 (6)	0.0156 (2)	
N2	0.34896 (7)	0.74941 (9)	0.22034 (6)	0.01294 (19)	
N3	0.32317 (7)	0.74721 (9)	0.13320 (6)	0.0136 (2)	
C1	0.13336 (8)	0.53784 (10)	0.31548 (7)	0.0133 (2)	
C2	0.17983 (8)	0.50147 (11)	0.39019 (7)	0.0144 (2)	
C3	0.27622 (8)	0.36991 (10)	0.30988 (7)	0.0141 (2)	
C4	0.23111 (8)	0.40222 (10)	0.23297 (7)	0.0129 (2)	
C5	0.17563 (8)	0.51489 (10)	0.22856 (7)	0.0120 (2)	
H5A	0.1231	0.5084	0.1824	0.014*	
C6	0.23963 (8)	0.61354 (10)	0.20620 (7)	0.0123 (2)	
C7	0.25677 (8)	0.66432 (10)	0.12467 (7)	0.0125 (2)	
C8	0.29982 (8)	0.67097 (10)	0.26530 (7)	0.0132 (2)	
H8A	0.3058	0.6580	0.3264	0.016*	
C9	0.21375 (9)	0.63618 (10)	0.03755 (7)	0.0141 (2)	
C10	0.11507 (9)	0.62914 (11)	0.02224 (8)	0.0162 (2)	
H10A	0.0751	0.6420	0.0688	0.019*	
C11	0.07515 (9)	0.60366 (12)	−0.05989 (8)	0.0182 (2)	
H11A	0.0082	0.5994	−0.0694	0.022*	
C12	0.13333 (9)	0.58421 (11)	−0.12869 (7)	0.0172 (2)	
C13	0.23124 (9)	0.58855 (12)	−0.11424 (8)	0.0186 (3)	
H13A	0.2712	0.5736	−0.1606	0.022*	
C14	0.27062 (9)	0.61488 (11)	−0.03141 (8)	0.0167 (2)	
H14A	0.3376	0.6183	−0.0219	0.020*	
C15	0.41077 (8)	0.83539 (10)	0.25672 (7)	0.0134 (2)	
C16	0.42022 (9)	0.93974 (11)	0.21407 (8)	0.0175 (2)	
H16A	0.3877	0.9529	0.1591	0.021*	
C17	0.47758 (10)	1.02438 (12)	0.25260 (9)	0.0223 (3)	
H17A	0.4849	1.0955	0.2234	0.027*	
C18	0.52447 (10)	1.00658 (12)	0.33343 (9)	0.0226 (3)	
H18A	0.5631	1.0654	0.3596	0.027*	
C19	0.51448 (9)	0.90238 (12)	0.37554 (8)	0.0190 (3)	
H19A	0.5464	0.8899	0.4308	0.023*	
C20	0.45806 (9)	0.81588 (11)	0.33746 (7)	0.0155 (2)	
H20A	0.4518	0.7442	0.3662	0.019*	
C21	0.23800 (9)	0.33188 (11)	0.15483 (7)	0.0150 (2)	
C22	0.17954 (12)	0.30966 (13)	0.00848 (9)	0.0288 (3)	
H22A	0.1412	0.3508	−0.0367	0.043*	
H22B	0.1510	0.2344	0.0186	0.043*	
H22C	0.2439	0.2991	−0.0106	0.043*	
C23	0.04876 (9)	0.61105 (11)	0.31701 (8)	0.0158 (2)	
C24	−0.05368 (10)	0.73192 (13)	0.23212 (9)	0.0244 (3)	
H24A	−0.0586	0.7705	0.1754	0.037*	
H24B	−0.0512	0.7897	0.2786	0.037*	
H24C	−0.1091	0.6823	0.2378	0.037*	
C25	0.34522 (9)	0.27246 (12)	0.32367 (8)	0.0195 (3)	
H25A	0.3937	0.2775	0.2807	0.029*	
H25B	0.3114	0.1992	0.3167	0.029*	
H25C	0.3756	0.2772	0.3825	0.029*	
C26	0.15698 (10)	0.53149 (12)	0.48159 (8)	0.0204 (3)	
H26A	0.1351	0.6114	0.4835	0.031*	
H26B	0.2139	0.5224	0.5205	0.031*	
H26C	0.1070	0.4803	0.5004	0.031*	
C27	0.14331 (11)	0.56823 (14)	−0.28286 (8)	0.0267 (3)	
H27A	0.1017	0.5606	−0.3358	0.040*	
H27B	0.1907	0.5066	−0.2810	0.040*	
H27C	0.1754	0.6429	−0.2830	0.040*	
H1N1	0.2957 (13)	0.4137 (15)	0.4321 (12)	0.027 (4)*	
H2W1	0.3944 (16)	0.828 (2)	0.0380 (14)	0.047 (6)*	
H2W2	0.4530 (18)	0.3690 (19)	0.5247 (14)	0.052 (6)*	
H1W1	0.4089 (18)	0.939 (2)	0.0070 (16)	0.063 (7)*	
H1W2	0.3581 (17)	0.345 (2)	0.5726 (15)	0.053 (6)*	

### Atomic displacement parameters (Å^2^)

**Table:**

	*U*^11^	*U*^22^	*U*^33^	*U*^12^	*U*^13^	*U*^23^
O1	0.0199 (5)	0.0257 (5)	0.0230 (4)	0.0020 (4)	0.0086 (4)	−0.0036 (4)
O1W	0.0300 (5)	0.0186 (5)	0.0229 (5)	−0.0027 (4)	0.0110 (4)	−0.0019 (4)
O2	0.0167 (4)	0.0197 (5)	0.0212 (4)	0.0060 (4)	0.0028 (3)	0.0014 (3)
O2W	0.0218 (5)	0.0177 (5)	0.0190 (4)	−0.0011 (4)	0.0007 (3)	0.0042 (3)
O3	0.0258 (5)	0.0186 (5)	0.0216 (4)	0.0060 (4)	0.0035 (4)	−0.0049 (3)
O4	0.0297 (5)	0.0170 (5)	0.0133 (4)	0.0042 (4)	0.0004 (3)	−0.0036 (3)
O5	0.0245 (5)	0.0303 (5)	0.0126 (4)	−0.0096 (4)	−0.0004 (3)	−0.0005 (3)
N1	0.0166 (5)	0.0170 (5)	0.0132 (4)	0.0014 (4)	0.0010 (4)	0.0005 (4)
N2	0.0147 (5)	0.0121 (5)	0.0119 (4)	−0.0009 (4)	0.0005 (3)	0.0001 (3)
N3	0.0153 (5)	0.0141 (5)	0.0115 (4)	−0.0002 (4)	0.0010 (3)	0.0005 (3)
C1	0.0135 (5)	0.0126 (5)	0.0142 (5)	−0.0020 (4)	0.0048 (4)	−0.0015 (4)
C2	0.0155 (5)	0.0134 (6)	0.0146 (5)	−0.0026 (4)	0.0044 (4)	−0.0006 (4)
C3	0.0140 (5)	0.0124 (6)	0.0162 (5)	−0.0012 (4)	0.0033 (4)	0.0000 (4)
C4	0.0130 (5)	0.0114 (5)	0.0147 (5)	−0.0010 (4)	0.0035 (4)	−0.0011 (4)
C5	0.0123 (5)	0.0117 (5)	0.0123 (5)	−0.0005 (4)	0.0022 (4)	−0.0001 (4)
C6	0.0131 (5)	0.0114 (5)	0.0126 (5)	0.0008 (4)	0.0023 (4)	0.0000 (4)
C7	0.0128 (5)	0.0113 (5)	0.0134 (5)	0.0008 (4)	0.0022 (4)	0.0001 (4)
C8	0.0150 (5)	0.0119 (5)	0.0130 (5)	−0.0005 (4)	0.0025 (4)	0.0004 (4)
C9	0.0165 (5)	0.0129 (6)	0.0130 (5)	−0.0013 (4)	0.0009 (4)	0.0016 (4)
C10	0.0154 (6)	0.0186 (6)	0.0148 (5)	0.0006 (5)	0.0027 (4)	0.0015 (4)
C11	0.0164 (6)	0.0206 (6)	0.0175 (5)	−0.0022 (5)	0.0000 (4)	0.0016 (4)
C12	0.0208 (6)	0.0173 (6)	0.0133 (5)	−0.0050 (5)	0.0002 (4)	0.0010 (4)
C13	0.0196 (6)	0.0229 (7)	0.0138 (5)	−0.0044 (5)	0.0044 (4)	−0.0016 (4)
C14	0.0150 (5)	0.0196 (6)	0.0155 (5)	−0.0034 (5)	0.0026 (4)	0.0003 (4)
C15	0.0126 (5)	0.0124 (6)	0.0153 (5)	−0.0008 (4)	0.0030 (4)	−0.0016 (4)
C16	0.0205 (6)	0.0156 (6)	0.0166 (5)	−0.0027 (5)	0.0027 (4)	0.0014 (4)
C17	0.0265 (7)	0.0173 (6)	0.0235 (6)	−0.0076 (5)	0.0037 (5)	0.0009 (5)
C18	0.0218 (6)	0.0218 (7)	0.0243 (6)	−0.0091 (5)	0.0023 (5)	−0.0043 (5)
C19	0.0155 (6)	0.0227 (7)	0.0187 (5)	−0.0025 (5)	0.0005 (4)	−0.0021 (5)
C20	0.0147 (5)	0.0151 (6)	0.0168 (5)	−0.0001 (4)	0.0020 (4)	−0.0001 (4)
C21	0.0152 (5)	0.0141 (6)	0.0161 (5)	−0.0024 (4)	0.0042 (4)	−0.0006 (4)
C22	0.0461 (9)	0.0240 (7)	0.0158 (6)	0.0082 (6)	−0.0015 (5)	−0.0069 (5)
C23	0.0149 (5)	0.0143 (6)	0.0183 (5)	−0.0019 (4)	0.0033 (4)	−0.0018 (4)
C24	0.0187 (6)	0.0235 (7)	0.0307 (7)	0.0075 (5)	−0.0007 (5)	−0.0011 (5)
C25	0.0203 (6)	0.0180 (6)	0.0203 (6)	0.0050 (5)	0.0023 (4)	0.0015 (4)
C26	0.0243 (6)	0.0227 (7)	0.0146 (5)	0.0020 (5)	0.0048 (4)	−0.0013 (4)
C27	0.0315 (7)	0.0350 (8)	0.0139 (5)	−0.0139 (6)	0.0034 (5)	−0.0020 (5)

### Geometric parameters (Å, º)

**Table:**

O1—C23	1.2129 (15)	C10—C11	1.3848 (16)
O1W—H2W1	0.89 (2)	C10—H10A	0.9500
O1W—H1W1	0.87 (3)	C11—C12	1.3957 (18)
O2—C23	1.3553 (15)	C11—H11A	0.9500
O2—C24	1.4408 (15)	C12—C13	1.3890 (18)
O2W—H2W2	0.98 (2)	C13—C14	1.3939 (16)
O2W—H1W2	0.90 (3)	C13—H13A	0.9500
O3—C21	1.2204 (16)	C14—H14A	0.9500
O4—C21	1.3507 (14)	C15—C16	1.3897 (17)
O4—C22	1.4364 (15)	C15—C20	1.3936 (15)
O5—C12	1.3709 (14)	C16—C17	1.3859 (18)
O5—C27	1.4324 (16)	C16—H16A	0.9500
N1—C3	1.3773 (15)	C17—C18	1.3894 (18)
N1—C2	1.3879 (16)	C17—H17A	0.9500
N1—H1N1	0.904 (18)	C18—C19	1.3853 (19)
N2—C8	1.3596 (15)	C18—H18A	0.9500
N2—N3	1.3683 (13)	C19—C20	1.3915 (17)
N2—C15	1.4207 (15)	C19—H19A	0.9500
N3—C7	1.3463 (15)	C20—H20A	0.9500
C1—C2	1.3570 (15)	C22—H22A	0.9800
C1—C23	1.4688 (17)	C22—H22B	0.9800
C1—C5	1.5192 (15)	C22—H22C	0.9800
C2—C26	1.5015 (16)	C24—H24A	0.9800
C3—C4	1.3630 (15)	C24—H24B	0.9800
C3—C25	1.5018 (17)	C24—H24C	0.9800
C4—C21	1.4616 (16)	C25—H25A	0.9800
C4—C5	1.5266 (16)	C25—H25B	0.9800
C5—C6	1.5129 (16)	C25—H25C	0.9800
C5—H5A	1.0000	C26—H26A	0.9800
C6—C8	1.3801 (15)	C26—H26B	0.9800
C6—C7	1.4194 (15)	C26—H26C	0.9800
C7—C9	1.4751 (15)	C27—H27A	0.9800
C8—H8A	0.9500	C27—H27B	0.9800
C9—C14	1.3897 (17)	C27—H27C	0.9800
C9—C10	1.4026 (17)		
			
H2W1—O1W—H1W1	105 (2)	C13—C14—H14A	119.4
C23—O2—C24	114.85 (10)	C16—C15—C20	120.56 (11)
H2W2—O2W—H1W2	116 (2)	C16—C15—N2	120.19 (10)
C21—O4—C22	116.13 (10)	C20—C15—N2	119.19 (11)
C12—O5—C27	117.00 (10)	C17—C16—C15	119.25 (11)
C3—N1—C2	123.24 (10)	C17—C16—H16A	120.4
C3—N1—H1N1	116.1 (11)	C15—C16—H16A	120.4
C2—N1—H1N1	120.4 (11)	C16—C17—C18	120.85 (12)
C8—N2—N3	111.77 (9)	C16—C17—H17A	119.6
C8—N2—C15	126.34 (10)	C18—C17—H17A	119.6
N3—N2—C15	121.40 (10)	C19—C18—C17	119.50 (12)
C7—N3—N2	104.64 (9)	C19—C18—H18A	120.3
C2—C1—C23	121.39 (11)	C17—C18—H18A	120.3
C2—C1—C5	119.64 (10)	C18—C19—C20	120.49 (11)
C23—C1—C5	118.56 (10)	C18—C19—H19A	119.8
C1—C2—N1	118.63 (10)	C20—C19—H19A	119.8
C1—C2—C26	126.88 (11)	C19—C20—C15	119.35 (12)
N1—C2—C26	114.48 (10)	C19—C20—H20A	120.3
C4—C3—N1	118.73 (11)	C15—C20—H20A	120.3
C4—C3—C25	126.67 (11)	O3—C21—O4	122.02 (11)
N1—C3—C25	114.59 (10)	O3—C21—C4	127.11 (11)
C3—C4—C21	120.58 (11)	O4—C21—C4	110.86 (10)
C3—C4—C5	119.21 (10)	O4—C22—H22A	109.5
C21—C4—C5	120.18 (10)	O4—C22—H22B	109.5
C6—C5—C1	109.73 (9)	H22A—C22—H22B	109.5
C6—C5—C4	110.43 (9)	O4—C22—H22C	109.5
C1—C5—C4	109.81 (9)	H22A—C22—H22C	109.5
C6—C5—H5A	108.9	H22B—C22—H22C	109.5
C1—C5—H5A	108.9	O1—C23—O2	122.43 (11)
C4—C5—H5A	108.9	O1—C23—C1	127.00 (11)
C8—C6—C7	104.47 (10)	O2—C23—C1	110.58 (10)
C8—C6—C5	124.81 (10)	O2—C24—H24A	109.5
C7—C6—C5	130.65 (10)	O2—C24—H24B	109.5
N3—C7—C6	111.46 (10)	H24A—C24—H24B	109.5
N3—C7—C9	119.67 (10)	O2—C24—H24C	109.5
C6—C7—C9	128.86 (11)	H24A—C24—H24C	109.5
N2—C8—C6	107.65 (10)	H24B—C24—H24C	109.5
N2—C8—H8A	126.2	C3—C25—H25A	109.5
C6—C8—H8A	126.2	C3—C25—H25B	109.5
C14—C9—C10	118.35 (10)	H25A—C25—H25B	109.5
C14—C9—C7	120.45 (11)	C3—C25—H25C	109.5
C10—C9—C7	121.20 (11)	H25A—C25—H25C	109.5
C11—C10—C9	120.89 (11)	H25B—C25—H25C	109.5
C11—C10—H10A	119.6	C2—C26—H26A	109.5
C9—C10—H10A	119.6	C2—C26—H26B	109.5
C10—C11—C12	119.97 (11)	H26A—C26—H26B	109.5
C10—C11—H11A	120.0	C2—C26—H26C	109.5
C12—C11—H11A	120.0	H26A—C26—H26C	109.5
O5—C12—C13	123.92 (11)	H26B—C26—H26C	109.5
O5—C12—C11	116.20 (11)	O5—C27—H27A	109.5
C13—C12—C11	119.87 (11)	O5—C27—H27B	109.5
C12—C13—C14	119.64 (11)	H27A—C27—H27B	109.5
C12—C13—H13A	120.2	O5—C27—H27C	109.5
C14—C13—H13A	120.2	H27A—C27—H27C	109.5
C9—C14—C13	121.26 (11)	H27B—C27—H27C	109.5
C9—C14—H14A	119.4		
			
C8—N2—N3—C7	−0.45 (13)	N3—C7—C9—C10	−130.42 (13)
C15—N2—N3—C7	−172.90 (10)	C6—C7—C9—C10	50.89 (18)
C23—C1—C2—N1	177.85 (11)	C14—C9—C10—C11	−1.15 (19)
C5—C1—C2—N1	−9.57 (17)	C7—C9—C10—C11	179.43 (12)
C23—C1—C2—C26	−2.5 (2)	C9—C10—C11—C12	0.2 (2)
C5—C1—C2—C26	170.03 (12)	C27—O5—C12—C13	−15.73 (19)
C3—N1—C2—C1	−16.56 (18)	C27—O5—C12—C11	164.95 (12)
C3—N1—C2—C26	163.79 (11)	C10—C11—C12—O5	−179.56 (12)
C2—N1—C3—C4	15.78 (18)	C10—C11—C12—C13	1.1 (2)
C2—N1—C3—C25	−163.19 (11)	O5—C12—C13—C14	179.27 (12)
N1—C3—C4—C21	−171.01 (11)	C11—C12—C13—C14	−1.4 (2)
C25—C3—C4—C21	7.82 (19)	C10—C9—C14—C13	0.80 (19)
N1—C3—C4—C5	10.87 (17)	C7—C9—C14—C13	−179.78 (12)
C25—C3—C4—C5	−170.29 (11)	C12—C13—C14—C9	0.5 (2)
C2—C1—C5—C6	−89.71 (13)	C8—N2—C15—C16	−148.56 (12)
C23—C1—C5—C6	83.08 (12)	N3—N2—C15—C16	22.74 (17)
C2—C1—C5—C4	31.85 (15)	C8—N2—C15—C20	28.37 (18)
C23—C1—C5—C4	−155.36 (10)	N3—N2—C15—C20	−160.34 (11)
C3—C4—C5—C6	88.64 (13)	C20—C15—C16—C17	0.22 (19)
C21—C4—C5—C6	−89.49 (12)	N2—C15—C16—C17	177.11 (12)
C3—C4—C5—C1	−32.51 (14)	C15—C16—C17—C18	−0.8 (2)
C21—C4—C5—C1	149.37 (11)	C16—C17—C18—C19	0.7 (2)
C1—C5—C6—C8	39.26 (15)	C17—C18—C19—C20	0.0 (2)
C4—C5—C6—C8	−81.93 (14)	C18—C19—C20—C15	−0.63 (19)
C1—C5—C6—C7	−144.34 (12)	C16—C15—C20—C19	0.50 (18)
C4—C5—C6—C7	94.47 (14)	N2—C15—C20—C19	−176.42 (11)
N2—N3—C7—C6	0.19 (13)	C22—O4—C21—O3	2.43 (18)
N2—N3—C7—C9	−178.72 (10)	C22—O4—C21—C4	−176.47 (12)
C8—C6—C7—N3	0.11 (14)	C3—C4—C21—O3	−3.0 (2)
C5—C6—C7—N3	−176.84 (11)	C5—C4—C21—O3	175.14 (12)
C8—C6—C7—C9	178.90 (12)	C3—C4—C21—O4	175.88 (11)
C5—C6—C7—C9	1.9 (2)	C5—C4—C21—O4	−6.03 (15)
N3—N2—C8—C6	0.53 (14)	C24—O2—C23—O1	−3.27 (17)
C15—N2—C8—C6	172.54 (11)	C24—O2—C23—C1	176.54 (10)
C7—C6—C8—N2	−0.38 (13)	C2—C1—C23—O1	−18.1 (2)
C5—C6—C8—N2	176.81 (11)	C5—C1—C23—O1	169.29 (12)
N3—C7—C9—C14	50.18 (17)	C2—C1—C23—O2	162.14 (11)
C6—C7—C9—C14	−128.52 (14)	C5—C1—C23—O2	−10.52 (15)

### Hydrogen-bond geometry (Å, º)

Cg1 and Cg2 are the centroids of the pyrazole (N2/N3/C6–C8) and benzene (C15–C20) rings, respectively.

**Table:**

*D*—H···*A*	*D*—H	H···*A*	*D*···*A*	*D*—H···*A*
N1—H1*N*1···O2*W*	0.904 (18)	2.001 (18)	2.9020 (14)	175.2 (15)
O1*W*—H2*W*1···N3	0.90 (2)	2.05 (2)	2.9449 (14)	174 (2)
O2*W*—H2*W*2···O1*W*^i^	0.98 (3)	1.84 (3)	2.7986 (15)	166 (2)
O2*W*—H1*W*2···O3^ii^	0.90 (3)	1.91 (3)	2.8074 (14)	174 (2)
O1*W*—H1*W*1···O2*W*^iii^	0.87 (3)	2.06 (3)	2.9274 (15)	178 (2)
C20—H20*A*···O1*W*^iv^	0.95	2.44	3.2980 (16)	151
C13—H13*A*···*Cg*2^v^	0.95	2.90	3.6957 (14)	142
C18—H18*A*···*Cg*1^vi^	0.95	2.63	3.3682 (15)	135
C25—H25*A*···*Cg*2^i^	0.98	2.87	3.7231 (14)	146
C27—H27*C*···*Cg*1^v^	0.98	2.62	3.5720 (17)	164

Symmetry codes: (i) −*x*+1, *y*−1/2, −*z*+1/2; (ii) *x*, −*y*+1/2, *z*+1/2; (iii) *x*, −*y*+3/2, *z*−1/2; (iv) *x*, −*y*+3/2, *z*+1/2; (v) *x*, −*y*+1/2, *z*−3/2; (vi) −*x*+1, *y*+1/2, −*z*+1/2.
